# Dr. Parry's Elements of Pathology and Therapeutics

**Published:** 1816-02

**Authors:** 


### *Dr. Parry's Elements of Pathology and Therapeutics.*

( Continued from page 49. )

HÆMORRHAGE.—As hæmorrhage frequently attends discharges of mucus, pus, and serum from inflamed parts, as the bronchia, liver, &c. it is considered by our author, as the topical effect of that increased momentum, forming a part of local inflammation. The same effect may happen from general plethora, as is shewn by the bloody urine following scarlatina, and sometimes measles; forming, in scarlatina, one link of a series of effects, of which articular inflammation and dropsy often constitute other links. Here anasarca or ascites being vicarious with hæmorrhage, affords new evidence of the nature of dropsy, as discussed at the close of last month's Review. Our author has more than once, seen obstinate and extensive anasarca cured by spontaneous hæmorrhage. Active hæmorrhage is always accompanied with phlogistic diathesis, and when the blood is discharged from the nose or viscera, it appears to be arterial, either from ruptured arteries or open mouthed capillaries.

Dr. Parry here mentions "a modification of purpura, exhibiting those appearances under the skin, which from their size and shape have been generally called petechiæ, maculæ, or vibices. They are usually flat, but sometimes considerably relieved above the rest of the skin. They are of different tints and shades of colour, from a pale red to a logwood purple, or even nearly the hue of a black currant. They do not become in any degree fainter from pressure, and are evidently ecchymoses or spots of extravasated blood. Their chief, but not only seat, is the upper or lower extremities. In most cases they have followed or accompanied pretty severe pain in the limbs, which has sometimes had the form of articular inflammation." Page 156.

Dr. Parry has published two cases in the 5th vol. of the Edinburgh Journal, and we ourselves have witnessed within these few weeks a similar disease, which embarrassed us much at the beginning.

A young Gentleman, after a hearty supper of oysters, was seized next day with bilious vomitings, head-ache, and considerable pyrexia. The fever continued for several days, with great determination to the head, notwithstanding strong cathartics were daily administered. About the sixth or seventh day, he was seized with violent pain in the small of the back, and in the region of the kidnies; soon after which, an eruption, similar to that above described, came out on the lower extremities, accompanied with excruciating pain in the limbs, and great tenderness and irritability on the surface; fever and restlessness still continued; and the intestinal discharges were invariably dark coloured and offensive. For three days, he took a grain of calomel and two of antimonial powder every three or four hours, which kept up a constant catharsis, and finally carried off the complaint. The appearance of these petechiæ would, we presume, a few years back, or even among our Brunonian brethren of the present day, have given origin to a very copious exhibition of bark and wine, to correct the putrescency of the humours and the debility of the solids.

Hæmatemesis and hemiplegia are brought forward by our author; the latter he has seen rapidly follow a violent palpitation of the heart, and was occasioned by a large extravasation of blood into the medullary substance of the brain, under which the patient lingered several weeks. In these cases of hemiplegia, and in many other hæmorrhages, the patient is, to all appearance, in such a state of previous good health, that one can hardly suspect phlogistic diathesis, or attribute the accidents to any other cause "than simple excessive momentum, acting perhaps, on vessels previously disposed to be so affected." Hæmorrhages may arise from general excessive momentum alone, as an exciting cause. Thus globules of blood are sometimes seen mixed with the perspired fluid in the axillæ of young persons after violent exertions; strong exercise, late hours, close rooms, or exposure to the intense heat of fires or sun will produce nasal hæmorrhage, and the same is produced by hard drinking in older persons.

"Sanguinis a naso fluxum, fere lethalem, Juveni, a coitu prima nuptiarum nocte sæpius repetito accidisse novi."

In respect to passive hæmorrhages, Dr. Parry seems a little puzzled, as there is seldom any indication of increased momentum.

"But if," says he, "a great degree of momentum be required to produce hæmorrhage in vessels little disposed, a slight degree will be sufficient in vessels which are strongly disposed. These, therefore, are the two states which constitute active and passive hæmorrhage." Page 162.

Dr. Parry regrets, that he has not seen a sufficient number of cases of sea scurvy, to enable him to decide whether, at a certain period of that disease, there be not excessive momentum that may ultimately produce those extravasations and passive hæmorrhages so characteristic of the disease. We have had some extensive opportunities of seeing scurvy, and Dr. Parry's reflections on the subject have recalled to our minds many circumstances favourable to his ideas. We always observed, that the first symptoms of sea scurvy broke out among those who were addicted to drunkenness, laziness, and gluttony, all which undoubtedly tend to produce general or local plethora, with consequent hæmorrhagic discharges. We observed on the other hand, that after great exertions of body and anxiety of mind, any sudden disappointment, as the escape of a prize, the discomfiture of an attack, or the like, gave origin to a formidable list of scorbutics, which extended even to those of active, sober, and cleanly habits. This we conceive, was owing to the sudden atony of the *extreme vessels* invariably following the application of the *depressing passions* to minds and bodies bordering on exhaustion from previous fatigue, and very fairly accounting for the hæmorrhages and extravasation in question. The petechial spots in typhus itself are usually the result of undue action of the heart upon skins suffering an accumulation of heat from various causes, as alcohol and other stimulating ingesta, and seldom appear where the treatment goes to regulate the balance of the circulation. The varicose state of the saphena, giving origin to hæmorrhage, is often certainly the consequence of mechanical obstruction to the return of the blood; but in many other cases, a diseased state of the coats of the vein is more probably the cause of the morbid dilatation, and of the hæmorrhage.

On the subject of hæmorrhoidal discharges, Dr. P. declines speaking, but refers to the illustrations of Mr. Abernethy. Page 166.

*Final Causes of Inflammation, Dropsy, and Hæmorrhage.*

" In the preceding pages, an attempt has been made to shew a coincidence of action or affection between inflammation, dropsy, and hæmorrhage, inasmuch as each of them is the consequence of excessive momentum of blood, whether relative or absolute."

These actions or affections *of the part* often remove the local malady, which in its turn frequently has a tendency to relieve the general momentum. Blood being the great stimulus to the heart's action, a certain fulness of the vessels, proportioned to the varying state of the system, is necessary for a due action of that organ. Thus excessive quantum increases, defective diminishes the action of the heart, as is evinced by the pulse in arterial and venous plethora following full living, " and the immediate change of that state by blood-letting." Page 169.

" These circumstances," says Dr. P. " render it probable, that one of the ends to be answered, in such cases, by the supervention of local inflammation, is, in various ways, to evacuate and soothe the constitution, which was before unduly stimulated by excessive vascular fulness." *Ib.*

In this way we partly conceive the salutary influence of gout in constitutions subject to excessive sanguineous momentum, and how other local affections supply the place of gout, as erysipelas, and various cutaneous affections. So also, hæmorrhages and hæmorrhoidal discharges prove vicarious of the same disease; thus our author has seen a patient accustomed to vernal gout, and missing the annual fit, have *erysipelas;* the next spring, *hæmorrhoids;* the following spring, a *fever,* cured by blood-letting; each with equal relief to the constitutional symptoms. The same effect precisely (in his experience) has been produced by anasarcous swellings of the lower extremities occuring at the gouty seasons. These circumstances then, shew, that constitutional errors of circulation are alleviated by local inflammation; hæmorrhage; and dropsy. So also increased action of the heart is relieved by dropsical effusions. Our author has seen a hectic pulse of 130 reduced in a few hours to 60, by the supervention of violent anasarca in the lower extremities.

" I have, indeed, so often known constitutional maladies suspended, and life evidently lengthened and rendered more comfortable, by the coming on of various dropsical effusions; and, on the contrary, so many persons suffer aggravations of disease, or even death, very shortly after the spontaneous disappearance of dropsy, that I cannot avoid considering that effusion as a salutary process, rather than as an actual disease." Page 171.

These pathological points are certainly of the utmost importance both in a speculative and practical view, inasmuch as they direct or sanction modes of treatment so active as to be either essentially beneficial on the one hand, or highly injurious on the other.

The coincidence of hæmorrhage with dropsy is not frequent; but Dr. P. has seen a long-continued and large hæmorrhage from the lungs, accompanied with hydrothorax, anasarca, and ascites, with a pulse at 136, all relieved together, and the patient restored to health as soon as, by digitalis, the pulse was reduced to 40 in the minute. Although dropsy often appears a consequence of hæmorrhage, Dr. P. thinks that it is rather from the *cessation* of hæmorrhage. Where the *latter* is accidental, but in some degree habitual, the dropsy arises either from a similar cessation, or from too sudden nutrition, both of which produce excessive plethora, often resulting from the means employed to arrest the disease and restore the strength!

*Simple excessive Determination, or Fulness of Blood.*

This is a very long and a very important section in our excellent author's work, and must receive proportionate attention.

Every accurate practitioner must have observed, that long after the subsidence of *actual* inflammation, gout, &c. in parts, an increased momentum of blood, accompanied by pain, swelling, or serous effusions, remained. Pains of various other parts appear to originate in this increased momentum, producing excessive impulse on them when in a state of susceptibility; and hence idiopathic pain in the animal frame can only be accounted for in this way (independent of "*inexplicable nervous sympathy*") "since pressure, bruising, tearing, cutting, stretching, suction, and probably all chemical operations, are mere modifications of that power." 178. One principal, almost one only objection to the doctrines of Dr. Parry, is his total neglect of the *nervous system.*

"He utterly, and for ever, disclaims all reliance on the *neurological* systems of pathology hitherto extant. He considers them as founded on principles which are either visionary or inapplicable, and which lead to practices tending equally to debase the moral character of mankind, to produce or perpetuate disease, and to discredit the medical profession." Preface, v.

This is too severe, and perhaps unjust. The nervous system must not be overlooked in pathology; for we are quite convinced, from attentive observation and reflection, that whether primarily or secondarily affected in disease, it bears as important a part as the vascular system; in short, that the *two systems* are so mutually dependent on, and connected with each other, that our views must be constantly directed to both, if we wish to practise with discrimination and success. But to return to our author.

"Pain itself has a tendency to diminish the action of the heart, and therefore that increased momentum or fulness of blood which produced it." 178.

Does not this very sentence of Dr. Parry's admit the mutual connexion we have been insisting on? Dr. Parry justly remarks, that we often see swelling, increased heat or redness, and occasionally all three, without actual inflammation, though these are the common characteristics laid down by nosologists. Since, however, the two states vibrate backwards and forwards into each other, so that what is one day a *determination* to a part, will the next be *inflammation*, there is evidence of one common condition in the two cases. These determinations produce different effects in different structures. In the serous and cellular textures, inflammation or effusion is very generally the result. Effusion, in some places, assumes peculiar appearances; viz. in the uvula, a semi-transparent elongation and swelling, &c.; when the increased determination is to the skin, heat, redness, and perspiration follow. Although a determination of blood to the nose from drink is frequently *coexistent*, Dr. P. thinks it not at all *connected* with hepatic affection, as suspected by Darwin and others. Dr. Parry here alludes to various anomalous, and indeed unaccountable pains and swellings, short of inflammation, which affect different parts of the human frame. Among these, we may instance that tenderness of the soles of the feet during the intervals of gout; those pains of the hypochondria in nervous females; the pain and soreness of the head in the same description of people, which Dr. Parry, perhaps with much truth, asserts to be peculiarly difficult of relief "under the false theory which dictates the usual treatment." 185. Another example is furnished by the rather sudden and painful swelling of one or both mammæ, alternating frequently with disorders of the head, and unconnected with that uneasiness or tumefaction preceding the menstrual periods. Our author has often seen "enlargements of the uterus with a sense of weight and bearing down, and sometimes with various discharges," yet unattended with fever, or any evidence of local inflammation, frequently disappear, either spontaneously, or under medical treatment. The knowledge of such circumstances ought to make us guarded in our prognosis on such occasions, since it is generally of an unfavourable kind. The prostate and other glands often undergo enlargement without inflammation or scirrhosity. The most remarkable disposition to enlargement without inflammation is evinced in the thyroid gland, constituting goitre or bronchocele.

"I have so often seen this swelling follow diseases of the heart, and other maladies, more especially those called nervous, such as epilepsy, &c. in which the blood is propelled, with excessive momentum, to the vessels of the head, and yet at the same time have observed such sudden augmentations and diminutions of the swelling, that I have suspected the gland itself to be intended as a diverticulum for blood disposed to flow with too great force to that important organ, the brain." 188.

Whether or not it be an accidental concatenation we know not; but in two decided cases of cardiac enlargement, now under our care, the thyroid gland is considerably enlarged. The liver and spleen are well known to suffer enormous turgescence, especially during the cold fits of ague, without inflammation, or any bad consequence. The vascular system of muscular parts also exhibits, on many occasions, these signs of turgescence; as, for instance, in the lower extremities previous to gouty paroxysm, &c. Speaking of those palpitations of the heart which so frequently affect females, when either chlorotic or labouring under strong nervous complaints, and which are excited by any muscular exertion, Dr. P. observes, that "the beating of the heart is usually felt at such a distance from its natural place, and there is often such a difficulty of lying on either side, that one cannot help concluding the heart to have suffered considerable enlargement."

"In such a case," says he, "I have known absorption of two of the ribs carried to a considerable extent; and pressure with the finger on the heart, through the yielding spot, produced great anxiety, and immediate disposition to syncope. Yet this patient recovered, and now enjoys tolerable health, thirty years after the period of my attendance." 191.

We quote this authentic and extraordinary instance, to shew that cardiac diseases should not be considered in quite so melancholy a point of view as they generally are; because we are convinced, from experience, that much may be done by regimen and medicines, even in the worst cases.

Few textures are more liable to increased determinations than mucous membranes, being exposed from situation to the constant influence of chemical and mechanical causes. Inflammation of these membranes is sufficiently common and sufficiently understood; but medical writers have not noticed, "that a state of excessive determination of blood to this membrane (that of the nose), though *without inflammation*, gives rise to some very common and important disorders." 195. Dr. P. has seen many instances of violent coryza without the least evidence of inflammation; sometimes by passing into a hot room—one by the internal use of hyoscyamus.

"In patients who are subject to spasmodic asthma, fits of that disorder often begin with a violent coryza, in which the eyes become red and watery, and all the symptoms of a cold in the head are observable. After a few days, or even hours, these symptoms suffer some degree of alleviation, and the malady proceeds to the bronchia, occasioning all the well-known symptoms of spasmodic asthma. What, then, is this state in the bronchia, but an affection of the mucous membrane of those cells, exactly similar to that which had previously existed in the same membrane in the nose?"

We think this a valuable observation. If it be said that asthma is a spasmodic affection depending on causes acting on the mind, &c. and periodical, Dr. P. answers, that the sense of suffocation in hydrothorax, and certain diseases of the heart, returns also at regular periods; while affections of the mind produce, aggravate, and renew gout, acute rheumatism, hæmorrhage, and various other disorders, to which no one thinks of assigning the name spasmodic. Again, what is termed spasmodic asthma is brought on by almost every thing that increases the action of the heart, and stimulates or fills the vessels of the mucous membrane itself; as intense heat, lightness of the air, exercise, full meals, stimulating drinks, certain effluvia—as those of hay, sealing-wax, ipecacuanha, &c.: whilst it is relieved by open bowels, heavy air, cold inhalations, and cooling drinks; diminishing as soon as a mucous secretion, or spitting of blood, relieves the turgescence of the vessels. These facts, Dr. P. thinks, are convincing proofs of such a preternatural fulness of the vessels of the mucous membrane of the bronchia, as to impede free inspiration, and to produce all the symptoms of spasmodic asthma. There can be little difficulty in understanding how a vascular fulness of the bronchial mucous membrane should produce dyspnœa by mere mechanical diminution of the tubes and cells, when a similar affection of the same membrane in the nose renders it sometimes absolutely impossible for us to respire through the capacious opening of the nostrils; and hence the absurdity, says Dr. P. of assuming asthma to be a nervous disease, produced by a *spasmodic* constriction of the air tubes.

This discussion on asthma clears the way for a knowledge of various other affections of mucous membranes, hitherto improperly termed spasmodic. Thus those strictures in the urethra, which are frequently influenced by mental causes, are probably owing to a turgescence of blood rather than to spasm.

Dyspepsia, Dr. Parry thinks, is a morbid fulness of vessels in the villous coat of the stomach, for the following reasons:—1st. Its symptoms are those of increased sensibility; (which is usually attended with, or produced by, vascular fulness) thus this organ suffers from a quantity of food that in health would produce no uneasiness. 2d. The cardialgia evidently arises from increased turgescence of blood. But here we would ask, how is it, that carbonate of ammonia and other stimulants are the surest means of relief in such cases? 3dly. When vomiting occurs in dyspepsia, when the stomach is void of food, the ejected fluid is merely an unusual quantity of the natural mucous secretion. 4thly. All the symptoms of dyspepsia, as flatus, heartburn, &c. exist in the greatest degree, in those cases which are followed by vomiting of blood, and are relieved by that discharge till a similar congestion of blood again takes place in the vessels of the stomach. 5thly. As females with obstructed menses are peculiarly liable to this state of dyspepsia, with bloody vomiting, so those who are regular in their catamenial discharges, suffer much less from dyspeptic symptoms *during* those periods, but relapse again in the intervals. A greater degree of this excessive determination then, would probably bring the malady within the limits of inflammation; instances of which, the author thinks he has seen. If, then, idiopathic dyspepsia be excessive vascular fulness of the villous coat of the stomach—if this may be produced by mental affections, and occasionally run within the limits of inflammation, we may conceive the process by which mental affections may be followed by scirrhosity, and subsequent ulceration; also, why the same may be produced by spirituous liquors, and mechanical injury. This discussion on dyspepsia may throw some light on the formation of strictures in the intestines, and also in the œsophagus. In common diarrhœa, an excessive determination of blood to the villous coat of the intestines probably takes place for the purpose of expelling the offending matter.

" I have," says Dr. P. " seen vehement evacuations of this kind continued, by strong purges, for months, and even years ; and yet, after death, dissection shewed the entire villous coat of the greater part of the intestinal canal still preternaturally turgid with blood." 210.

We do not doubt it: — the " strong purges" were sufficient to keep up a very constant determination to the bowels; but might not the plethora have been diverted to the skin in such cases, by diaphoretics? or, obviated by abstemiousness and venæsection? This is corroborated by Dr. Parry himself.

" In a boy, seven years of age, there were such motions as I have described (copious and loose) with fever and almost total insensibility. He had, in vain, for sixteen days, tried all the most approved *aperients*, when *six ounces of blood* from the temporal artery, and repeated next day, immediately brought the stools to their natural state, and the patient to convalescence." 211.

Dr. P. gives us some interesting remarks on those pulsations which we sometimes feel in the abdomen, occasioning apprehensions of aneurism. In all persons not very fat, the pulse of the aorta can easily be felt, while lying on the back, if strong pressure be made a little to the left of the median line, about half way between the navel and scrobiculus cordis; in some instances, the pulsation is painfully perceived by the patient himself. In many cases of this kind, particularly in nervous patients, this sense of pulsation is merely the effect of preternatural action of the heart; in others, of the pressure of some hard substance on the descending aorta, determining a disproportionate quantity of blood to the head, " and giving to the hand placed on the abdomen, and sometimes even to the eye, the appearance of a beating so near the surface, as to lead inexperienced observers to conclude that the aorta is morbidly dilated." We are not ashamed to say, that we were once deceived in this way, and our prognosis turning out so wrong, has made us extremely careful since, in ascertaining the true state of the parts. The most common causes are collections of fæces in the colon, requiring repeated and active purgatives, which must bring away almost incredible discharges of stercoraceous matter before the aortal pulsation subsides.

In many cases of dyspepsia, the affection of the stomach is only secondary, while the primary disorder exists in the colon; the villous coat of which seems to be affected with morbid sensibility, unspeakable uneasiness, burning heat, and all those other circumstances which have been described as occurring in the stomach. This state frequently runs into inflammation, and lays the origin of strictures in that intestine.

Fluor albus and gleet are brought forward by Dr. P. as also affording examples of simple excessive determination of blood to mucous membranes. To this is attributed a common species of deafness, described by Dr. James Sims, and usually called nervous, depending on obstruction of the Eustachian tube.

"The patient can hear well when the tube is distended, by strongly blowing, with the nose, mouth, and cheeks closely shut."

Our author has known it removed by the supervention of hepatitis, and of hemiplegia; returning as these diminished; also entirely to cease, in two instances, forty-eight hours before death; and thirdly, completely cured for more than a year of the remainder of life, by an accidental hæmorrhage from the humeral artery.

The following are instances of increased determinations of blood producing secretions or excretions, morbid or natural. 1st. Spontaneous inordinate ptyalism is often produced by the influence of the mind, for it is frequently the result of an excessive attention to the discharge, or constant attempts to eject it, under the erroneous impression that it is excrementitious. Diabetes will probably be admitted as the effect of an excessive determination of blood to the renal arteries, since the secretion of urine is preternaturally increased; and dissection shews, that the kidnies are preternaturally vascular.

The turgescence of the genital organs in man and in animals is adduced by our author as an illustration of salutary, or at least natural determination of blood to certain parts, intended to be followed by increased secretion or excretion. So are the menstrual periods in women, which are generally preceded by pains in the loins, owing, in a great degree, to turgescence of the hypogastric arteries, and which disappear when the natural discharge is fully established. The progress of sanguineous momentum is beautifully illustrated by the phænomena of ephemeral fever. Here, at the commencement, the skin and extremities are cold, pale, shrunk, and in some degree benumbed, while the pulse is weak and soft. By degrees, as the action of the heart increases, the impetus of the blood is augmented, first to the head, and then to the trunk. At length, the extremest parts feel the influence of the vital current, and then perspiration breaks out as after violent exercise, diminishing the impetus and bringing relief to all its effects.

The same process frequently occurs to one part, or certain parts only, of the body, as the arm or both legs, which go through regular fits of what may be termed ague, beginning with preternatural coldness, and proceeding through excessive heat to termination by sweating. Of this progress of increased impetus, there is, in many women, an exemplification during menstruation.

"One or two days previously to that period, they suffer a violent pain and weight in the head, accompanied with flushing and heat of the forehead and cheeks, occasionally with sickness. In this state the pulsation of the carotids is strong; the feet are often cold, and the general symptoms are those of slight sick head-ache. In the course of one or two days, the head-ache becoming better, the back and loins begin to suffer aching pain; and this is a prelude to the natural discharge, which in a few hours appears; and then, the feet having previously become warm, all the uneasy feelings soon vanish." 223.

Nervous women often suffer sudden determinations to the skin of the face, and sometimes the greater part of the body. It is attended with a flushing and great heat of the skin, and in an instant succeeded by sweating; after which, the skin becomes cold, and the action of the heart is diminished, often in an undue degree. In the latter case, some faintness ensues. All the steps of the process are performed in a very rapid manner, and are called by the vulgar, in the author's part of the country, the hot blooms.

Dr. Parry hazards a conjecture, that fungus hæmatodes itself is occasioned, or at least accompanied, by an increased momentum of blood determining its specific nature.

We shall conclude the review of this important work in our next number, and hope that our final analysis will be the most interesting of the three. The length to which we have carried our *analysis*, and the paucity of *critical* interruption which the reader has met with, are sufficient proofs of the degree of importance which we attach to the labours of Dr. Parry.

